# Development of a Novel, Non-Invasive Saliva Sampling Method for the Detection of Bovine Respiratory Viruses

**DOI:** 10.3390/vetsci12070637

**Published:** 2025-07-03

**Authors:** Simona Baumann, Belinda Euring, Maxi Harzer, Mandy Eibisch, Andrea Lindner, Thomas W. Vahlenkamp, Kristin Heenemann

**Affiliations:** 1 Institute of Virology, Centre for Infectious Diseases, Faculty of Veterinary Medicine, Leipzig University, An den Tierkliniken 29, 04103 Leipzig, Germany; belinda.euring@vetmed.uni-leipzig.de (B.E.); maxi.harzer@vetmed.uni-leipzig.de (M.H.); vahlenkamp@vetmed.uni-leipzig.de (T.W.V.); kristin.heenemann@vetmed.uni-leipzig.de (K.H.); 2 BioCheck—Laboratory for Veterinary Diagnostics and Environmental Hygiene GmbH, Mölkauer Straße 88, 04288 Leipzig-Holzhausen, Germany; m.eibisch@biocheck-leipzig.de (M.E.); a.lindner@biocheck-leipzig.de (A.L.)

**Keywords:** bovine respiratory disease (BRD), respiratory viruses, non-invasive diagnostics, saliva sampling method

## Abstract

Bovine respiratory disease (BRD) is a major cause of economic loss and impaired animal welfare worldwide, especially in calves. It is a multifactorial disease complex caused by the interaction of viral and bacterial pathogens, environmental stressors and the host immune response. It has to be controlled by preventive measures and rapid pathogen detection. Current diagnostic methods are often time-consuming and impose a high burden on animals during sampling. In this study, we established a novel, non-invasive sampling system that allows the monitoring of viruses associated with BRD based on saliva samples. We selected five farms with animals displaying various degrees of respiratory symptoms. Cows and calves were sampled using the saliva sampling system based on cotton rolls. Additionally, deep nasal swabs were taken for comparison. The samples were tested for viral BRD pathogens using polymerase chain reactions (PCRs). The study showed that saliva is a suitable sample material for the detection of bovine respiratory viruses. Important viruses such as Bovine Respiratory Syncytial Virus, Bovine Parainfluenza Virus 3, Bovine Adenovirus, Bovine Coronavirus and Bovine Rhinitis A and B Virus were detected in saliva samples. While deep nasal swab samples showed slightly higher sensitivity for some viruses, other viruses were detected only in saliva. This highlights the complexity of BRD diagnosis and the limitations of single-site sampling. The introduction of this simple, non-invasive sampling method represents a major step forward in protecting animals and advancing welfare standards in the cattle industry.

## Simple Summary

Bovine respiratory disease is a commonly diagnosed health problem in calves. In addition to bacteria, several viruses contribute to the disease, highlighting the necessity for accurate differential diagnosis. The leading sampling method is invasive and distressing for the animals, requiring them to be restrained to collect deep nasal swabs. Therefore, our current study, carried out on five farms with animals showing respiratory symptoms, developed a new saliva sampling system based on cotton rolls. The saliva sampling system was hung in the stalls and the animals were free to chew on the materials. Deep nasal swabs were taken from the sampled animals as the gold standard for method comparison. Saliva showed to be a suitable sample material for the detection of respiratory viruses in cattle. Important viruses involved in the development of the respiratory disease could be detected. Calves in particular have shown great curiosity and interest in the established saliva collection system and it was possible to obtain a sufficient amount of saliva. The investigation of saliva obtained by the non-invasive sampling method has been shown to be suited for the detection of bovine respiratory viruses.

## 1. Introduction

Bovine respiratory disease (BRD) is one of the most significant health issues in cattle populations worldwide, leading to great economic losses and animal suffering [1]. Treatment of each calf with BRD has been calculated to cost USD 42.15, underscoring the importance of early diagnosis and prevention [2]. BRD is a leading cause of morbidity and mortality in feedlot cattle [3], affecting mostly young cattle and pre-weaned calves [4].

BRD is a complex and multifactorial syndrome, with different aspects contributing to the development of the disease. Its outcome is influenced by multiple factors such as viral and bacterial infections, the host’s genetics, immune response and microbiome, as well as environmental conditions and management practices, including stressors. Predisposing factors are required to induce a transient state of immunosuppression, allowing infectious agents to infect and colonize the host. In most cases, viral infections and/or environmental stressors weaken the immune system, enabling bacteria to access the lower respiratory tract and cause pneumonia [1]. Bacterial and viral infections can be promoted by management issues, such as overcrowding, weaning or dehorning [1,5]. Other important predisposing factors are environmental conditions, e.g., sudden temperature and climate changes, and long-distance transport. Hence, BRD is also described as shipping fever [6].

Many bacteria and viruses have been described as the cause of BRD or are associated with the disease. It is therefore considered to be a polymicrobial disease with a high frequency of coinfections [4]. Primary viral infections are assumed to be a prerequisite for the development of BRD [7]. Bovine Respiratory Syncytial Virus (BRSV; *Pneumoviridae*), Bovine Parainfluenza Virus 3 (BPIV3; *Paramyxoviridae*), Bovine Alphaherpesvirus 1 (BoAHV1; *Orthoherpesviridae*) and Bovine Coronavirus (BoCV; *Coronaviridae*) have been well-known viral BRD agents for years. Using next-generation sequencing, other viruses, such as Bovine Adenovirus (BAdV; *Adenoviridae*), Influenza D Virus (IDV; *Orthomyxoviridae*), Influenza A Virus (IAV; *Orthomyxoviridae*), Bovine Rhinitis A Virus (BRAV; *Picornaviridae*) and Bovine Rhinitis B Virus (BRBV; *Picornaviridae*), have recently been recognized as contributors in BRD [4,8,9]. Their involvement in the BRD process is part of current research [9,10]. Table 1 provides an overview of viruses involved in BRD. The main bacterial pathogens of BRD are *Mannheimia haemolytica*, *Pasteurella multocida*, *Histophilus somni* and *Mycoplasma bovis* [4,5]. Other opportunistic bacteria, including *Trueperella pyogenes*, *Mycoplasma bovirhinis* and *Bibersteinia* spp., may also contribute to the disease, particularly in chronic or immunocompromised cases [11].

The most common clinical signs of BRD are a combination of increased body temperature, cough, nasal discharge and dyspnea [12].

Various sampling techniques of the respiratory tract of calves and cattle are described for the diagnosis of BRD. They include nasopharyngeal swabs, transtracheal washes and non-/endoscopic bronchoalveolar lavages. The sampling site is of great significance for interpreting the test results [13]. During sampling, animals must be restrained to ensure safe and effective collection of sample material. In practice, nasopharyngeal swabs are commonly used for diagnosis, as they are simple to use and the results are consistent with the tracheal and bronchial samples [14].

In this study, saliva was investigated as sample material for the targeted detection of respiratory viruses. To date, only a limited number of studies have examined bovine saliva samples in this context. Bovine Viral Diarrhea Virus (BVDV), for example, has previously been identified in saliva using reverse transcription polymerase chain reaction (RT-PCR) [15]. Our aim was to develop a new sampling system for cattle to collect saliva in a stressless environment. The animals should interact with the sampling system voluntarily and without fixation or external influence from the veterinarian. Using saliva for diagnostics as a non-invasive sample material promotes animal welfare and improves farm conditions.

## 2. Materials and Methods

### 2.1. Ethical Statement

This study was approved by the Ethics Committee of the Faculty of Veterinary Medicine, Leipzig University (EK 07/2022).

### 2.2. Sample Collection

The samples were collected by a veterinarian between May 2023 and April 2024. A total of five dairy cattle farms in Saxony, Saxony-Anhalt and Brandenburg were included in the study. Cattle were selected for sampling based on respiratory symptoms, such as fever, nasal discharge and lung sounds. In some cases, animals without clinical signs of BRD were included when sampling was intended to determine the pathogens circulating on the farm.

The animals were sampled as follows: cows as individuals, cows in a group, calves as individuals and calves in a group. Table 2 shows the planned number of samples for each farm.

Two different sample matrices, including deep nasal swabs and saliva, were obtained from each group. The cows were sampled using a 60 cm long nasal swab (Erich EYDAM, Kiel, Germany), while a shorter, flocked nasal swab with Universal Transport Medium (UTM™) system (Mast Diagnostica, Reinfeld, Germany) was used for the calves. To obtain fresh nasal mucosal cells, the deep nasal swabs were inserted deep into the ventral nasal passages.

A newly developed sampling system based on cotton rolls was used to collect saliva. The cotton rolls were threaded onto a thin, tear-resistant string and then hung in the barns so that the animals could chew on them independently. The cotton rolls included in the Salivette^®^ (Sarstedt, Nümbrecht, Germany) were used. The saliva sampling system has been developed separately for use with cows and calves, as previous testing has shown that cattle acceptance varies depending on age.

**Figure 1:**
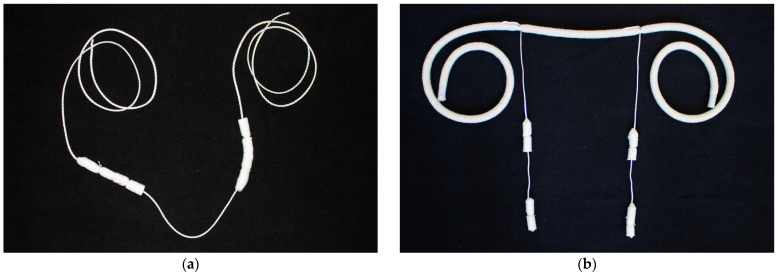
Newly developed saliva sampling system separately for cows (**a**) and calves (**b**). Four cotton rolls were threaded on a string and were hung in the barns, so that the animals could chew on it.

The cow saliva sampling system consisted of four cotton rolls threaded lengthwise on a string. To increase acceptance, the cotton rolls were wrapped in hay and then tied with string. For calves, we developed a saliva sampling system that also consisted of four cotton rolls. Two cotton rolls each were threaded onto a thin string, which was then tied to a thicker string. The sampling systems were sterilized in an autoclave to prevent contamination during preparation. Both saliva sampling systems are shown in Figure 1 below.

Cows were sampled as individuals with the saliva sampling system placed in front of them on the feeding table. It was attached to the feed fence to the left and right of the cow using the long string so that it could only be taken into the mouth of the targeted animal. Individual calves were sampled in their pens while they were still individually housed. The saliva sampling system was attached to the bars of the box and left in place for several minutes. For the group diagnosis of cows and calves, the respective saliva sampling systems were placed near the drinking trough. Depending on the size of the herd, one or two saliva sampling systems were added to the group, which were then pooled in the laboratory. For comparison, the deep nasal swabs were taken from the sampled individuals and five randomly selected animals from the groups, which were later pooled in the laboratory.

The samples were brought to the laboratory within 6 h, where they were processed and stored at −80 °C degrees. To obtain the saliva, the cotton rolls were placed individually in the corresponding Salivette^®^ (Sarstedt, Nümbrecht, Germany) and centrifuged (5 min, 2500× *g*). Next, 2.5 mL Phosphate-Buffered Saline (PBS) was added to each deep nasal swab (Erich EYDAM, Kiel, Germany) of the cows, which was then used for further analyses. Universal Transport Medium™, in which the calf deep nasal swabs were transported, was used directly for the extraction.

### 2.3. RNA/DNA Extraction and PCR Tests

Viral RNA and DNA were extracted with a commercially available kit (Farm 1: IndiMag Pathogen Kit, INDICAL BIOSCIENCE, Leipzig, Germany and Farm 2–5: QIAamp Viral RNA Kit, QIAGEN, Hilden, Germany), according to the manufacturer’s instructions. The concentration of DNA was measured by using NanoDrop (Thermo Fisher Scientific, Waltham, MA, USA). Each sample, whether a deep nasal swab or saliva, was treated in the same way.

Various PCR and RT-PCR tests were carried out for the different viral pathogens. All samples were tested for BAdV, BoHV-1, BRSV, BPIV3, IAV, IDV and BoCV, as described in previous studies [8,16,17,18,19,20,21,22]. Family-specific PCRs were used for the detection of BAdV and BoHV-1, allowing comprehensive identification of different types of BAdV and Bovine Herpesvirus (BoHV). New primers were designed and RT-PCR protocols were established to analyze samples for BRAV and BRBV. A detailed overview of all primers and PCR kits used is shown in Table 3.

The PCR products were prepared with DNA Gel Loading Dye (Thermo Fisher Scientific, Waltham, MA, USA) and then applied to a 2% agarose gel for electrophoresis.

### 2.4. Development of RT-PCRs for BRAV and BRBV

To design unique primer pairs for BRAV and BRBV, several described genome sequences were aligned using the software MEGA X (Version 10.1.8) and conserved regions were identified. Table 4 shows the primer sets designed for BRAV and BRBV.

A one-step nested RT-PCR protocol was established for BRAV, according to the manufacturer’s instructions. For the first step, a tube was prepared with a total volume of 25 μL, containing 0.25 μL of F and R 2 primer (each 100 μM), 4 μL of RNase-free double-distilled H_2_O (ddH_2_O), 12.5 μL of buffer, 2.0 μL of magnesium sulfate (5mM), 0.5 μL of SuperScript III RT/Platinum *Taq* Mix (Thermo Fisher Scientific, Waltham, MA, USA), 0.5 μL of RiboLock RNase Inhibitor (Thermo Fisher Scientific, Waltham, MA, USA) and 5 μL of the extracted sample. The following thermal profile was used: 60 °C for 1 min; 55 °C for 30 min; 94 °C for 2 min; 45 cycles of 94 °C for 5 s, 56 °C for 30 s and 68 °C for 20 s; and finally 68 °C for 5 min. For the second step, the reaction mixture, with a final volume of 25 μL, contained 0.25 μL of the F and R 1 primer (each 100 μM), 0.5 μL of dNTPs, 19.4 μL of RNase-free ddH_2_O, 2.5 μL of buffer, 1.0 μL of MgCl_2_ (50 mM), 0.1 μL of Platinum *Taq* DNA Polymerase (Thermo Fisher Scientific, Waltham, MA, USA) and 1 μL of the respective product from the first step. The following thermal profile was used: 94 °C for 2 min; 45 cycles of 94 °C for 30 s, 56 °C for 30 s and 72 °C for 20 s; and finally 72 °C for 5 min.

For BRBV, a one-step RT-PCR protocol was established with a total volume of 25 μL. The reactions consisted of the same reagents as in the first step of the protocol for BRAV, except for the primers and RNase-free ddH_2_O. Then, 1 μL of each primer (10 mM) and 2.5 μL of RNase-free ddH_2_O were added to the reaction. After 60 °C for 1 min, 55 °C for 30 min and 94 °C for 2 min, the reactions were cycled 45 times as follows: 94 °C for 15 s, 54 °C for 30 s and 68 °C for 20 s. At the end, the final elongation step was performed at 68 °C for 5 min.

The specificity of the two RT-PCR protocols was confirmed by testing additional Enteroviruses of the family *Picornaviridae*. To determine the sensitivity, specific plasmids were constructed using the CloneJET PCR Cloning Kit (Thermo Fisher Scientific, Waltham, MA, USA). A detection limit of 5 copies per reaction was determined for BRAV and BRBV.

### 2.5. Sequencing and Sequence Analysis

DNA fragments were purified from the agarose gel with an extraction kit (GeneJET Gel Extraction Kit, Thermo Fisher Scientific, Waltham, MA, USA) and sent to Microsynth Seqlab GmbH for Sanger sequencing. The resulting sequences were analyzed using GenBank, the database from the National Library of Medicine (NCBI). The Basic Local Alignment Search Tool (BLAST) was used to align the resulting sequences to the GenBank sequences.

### 2.6. Data Analysis

Data display was performed with GraphPad Prism (Version 10.4.1).

## 3. Results

Deep nasal swabs and saliva samples were collected from all five farms, as displayed in Table 5. Individual animals did not always interact with the saliva sampling system. Therefore, we did not obtain the planned number of samples from each farm. On farms 2 and 3, the calves in the groups did not chew on the cotton rolls, so we were unable to obtain saliva samples there. As the aim of this study was to collect saliva samples in a stressless environment, we did not restrain the animals for collection but allowed them to chew on the cotton rolls independently.

The cow groups consisted of at least 50 animals and the calf groups of at least 15 animals, with the exception of farm 5, where smaller calf groups were present. Due to the reduced group size, two separate calf groups were sampled on this farm. Instead of testing five calves, three calves from each of the two groups were comparatively tested using a deep nasal swab.

### 3.1. Virus Detection in Deep Nasal Swabs

**Figure 2:**
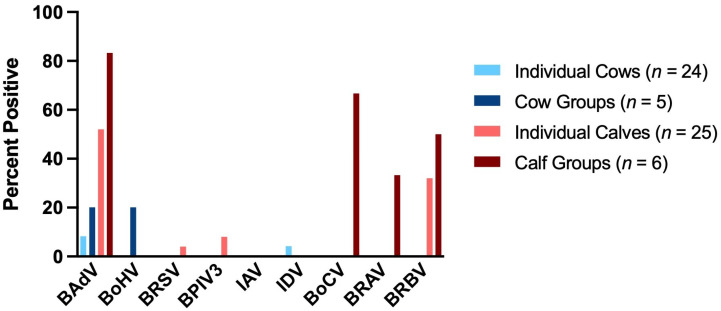
Detection rates (%) of respiratory viruses in deep nasal swabs collected from individual cows (*n* = 24; light blue), cow groups (*n* = 5; dark blue), individual calves (*n* = 25; red) and calf groups (*n* = 6; dark red).

As shown in Table 5, a total of 49 deep nasal swabs were collected from individual animals, 24 from cows and 25 from calves. A total of 11 pooled samples of deep nasal swabs were collected during the sampling of the animal groups, 5 from cow groups and 6 from calf groups. The results of the PCR and RT-PCR tests for viral pathogens are shown in Table 6 and Figure 2.

BAdV (35.0% in total, *n* = 60) was the most frequently detected virus in deep nasal swabs. With 52.0% in individual calves (*n* = 25) and 83.33% in groups (*n* = 6), it was highly prevalent in calves but was also found in cows. BoHV (20.0% in cow groups, *n* = 5) and IDV (4.17% in individual cows, *n* = 24) were each detected once in cows. IAV was not found in any of the samples (0.0%, *n* = 60). BRSV (4.0%, *n* = 25) and BPIV3 (0.8%, *n* = 25) were detected only in individually sampled calves. BoCV (66.67%, *n* = 6), BRAV (33.33%, *n* = 6) and BRBV (50.0%, *n* = 6) were frequently detected in the sampled calf groups. BRBV (32.0%, *n* = 25) was also detected in individually sampled calves.

### 3.2. Virus Detection in Saliva Samples

**Figure 3:**
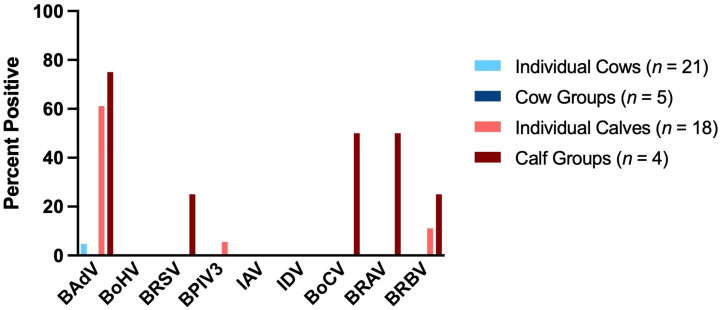
Detection rates (%) of respiratory viruses in saliva samples collected from individual cows (*n* = 21; light blue), cow groups (*n* = 5; dark blue), individual calves (*n* = 18; red) and calf groups (*n* = 4; dark red).

As shown in Table 5, a total of 39 saliva samples were collected from individual animals, 21 from cows and 18 from calves. A total of nine pooled samples of saliva were collected during the sampling of the animal groups, five from cow groups and four from calf groups. The results of the PCR and RT-PCR tests for viral pathogens are shown in Table 7 and Figure 3.

BAdV was the most frequently detected virus in the saliva samples with 31.25% (*n* = 48). It was the only virus detected in an individual cow (4.76%, *n* = 21). No other viruses were detected in the saliva of the cows. In the saliva samples of individuals and groups of calves, BAdV was highly prevalent with 61.11% (*n* = 18) and 75.0% (*n* = 4), respectively. BoHV, IAV and IDV could not be detected in the calves’ saliva. BRSV and BPIV3 were detected once in in a group of calves (25.0%, *n* = 4) and once in an individual calf (5.56%, *n* = 18). In half of the calf groups, BoCV and BRAV were detected (each 50.0%, *n* = 4). BRBV was detected in saliva samples in one calf group (25.0%, *n* = 4) and in two of the 18 individually sampled calves (11.11%, *n* = 18).

### 3.3. Sequencing Results

As the adenovirus and herpesvirus PCRs are family-specific PCRs, the sequencing results were of particular interest here. In around 230 bp, all sequenced products of the adenovirus PCR showed a homology of at least 99.1% to Bovine Adenovirus 3 (BAdV-3; according to the reference sequence NCBI-Acc.-No.: JN381195.1 and NC_001876.1). The partial genome sequences have been deposited in the National Center for Biotechnology Information under GenBank accession numbers PV217158, PV217159, PV217160, PV217161, PV217162, PV217163, PV217164, PV217165, PV217166, PV217167, PV217168, PV217169, PV217170, PV217171, PV217172, PV217173, PV217174, PV217175 and PV217176.

Herpesvirus was detected in one deep nasal swab of a cow. The sequencing of the PCR product showed in 116 bp a homology of 100.0% to Bovine Lymphotropic Herpesvirus (according to the reference sequence NCBI-Acc.-No.: AY237374.1), currently renamed as Bovine Gammaherpesvirus 6 (BoGHV6). The partial genome sequence has been deposited in the National Center for Biotechnology Information under GenBank accession number PV169275.

### 3.4. Comparison of Deep Nasal Swabs and Saliva Samples

To test the comparability of deep nasal swabs and saliva as sample matrices for respiratory viruses in cattle, only samples from animals or groups from which both matrices could be obtained were included. That was a total of 38 individual animals (20 cows and 18 calves) and 9 groups (5 cow and 4 calf groups), which were considered separately. For each virus, the number of paired deep nasal swabs and saliva samples with matching results was analyzed. This was performed by dividing the sample pairs into the following groups:

- Positive result in deep nasal swab and saliva sample (+/+);
- Negative result in deep nasal swab and saliva sample (−/−);
- Positive result in deep nasal swab and negative result in saliva sample (+/−);
- Negative result in deep nasal swab and positive result in saliva sample (−/+).

The percentage results are shown in Table 8 for the individual animals (*n* = 38) and for the groups (*n* = 9). To obtain the percentage of sample pairs that gave the same results, the respective percentages were summed and the results for each virus are also shown in Table 8.

For the individual animals, the highest number of positive results in deep nasal swabs and saliva samples was observed with 26.32% (*n* = 38) found for BAdV, followed by BRBV (5.26%, *n* = 38). A certain percentage of BAdV (7.89%), BRSV (2.63%), BPIV3 (5.26%), IDV (2.63%) and BRBV (15.79%, *n* = 38) could only be detected in deep nasal swabs and not in saliva samples. In some of the sample pairs, BAdV (5.26%) and BPIV3 (2.63%, *n* = 38) could only be detected in saliva samples and not in deep nasal swabs. Only negative results for BoHV, IAV, BoCV and BRAV were found in both deep nasal swabs and saliva samples (each 100.0%, *n* = 38).

Positive results in both sample matrices were detected in three groups for BAdV and BoCV (each 33.33%, *n* = 9), in two groups for BRAV (22.22%, *n* = 9) and in one group for BRBV (11.11%, *n* = 9). In one of the groups, BAdV, BoHV, BoCV and BRBV (each 11.1%, *n* = 9) could only be detected in the deep nasal swab and not in saliva samples. BRSV was detected in the saliva sample and not in the deep nasal swab in one group (11.1%, *n* = 9). In both matrices, the detection of BPIV3, IAV and IDV (each 100.0%, *n* = 9) was impossible.

**Figure 4:**
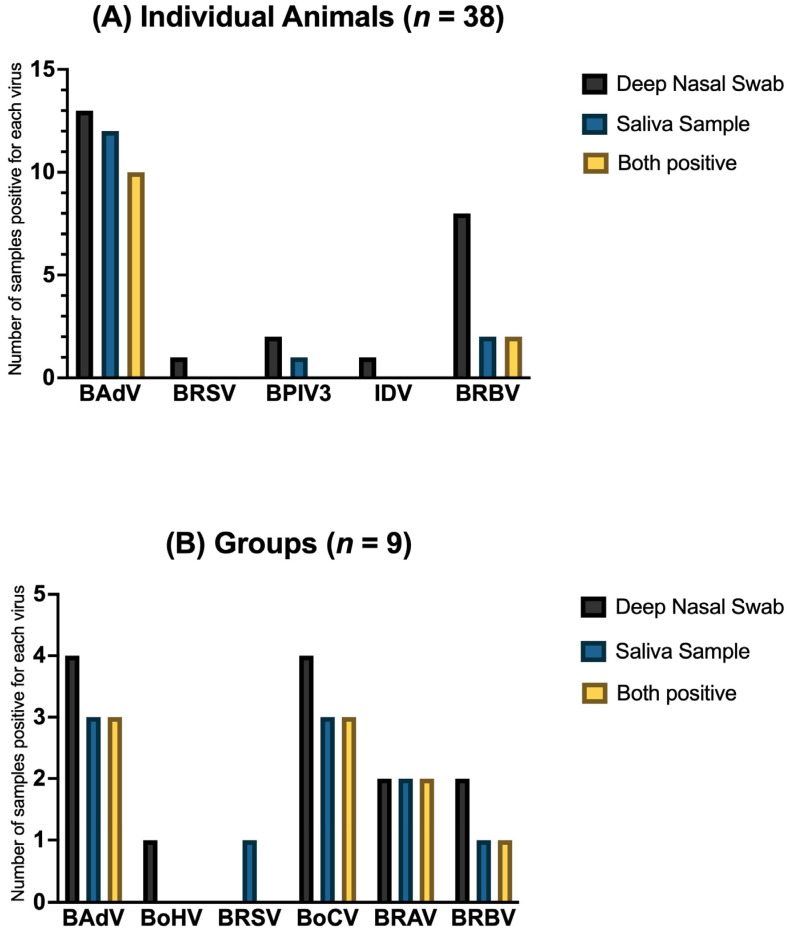
The number of positive samples for respiratory viruses in (**A**) individual animals (*n* = 38) and in (**B**) groups (*n* = 9), divided into sample matrices: deep nasal swabs (black) and saliva samples (blue). The number of positive samples for both matrices is shown in yellow. Only sample pairs where both matrices were available were included.

A comparison of the positive results in the two matrices is shown in Figure 4 for each virus. Only sample pairs where both matrices were available were included.

## 4. Discussion

This study demonstrates that saliva is a suitable material for the detection of different respiratory viruses in cattle. BAdV, BRSV, BPIV3, BoCV, BRAV and BRBV were successfully detected in the saliva samples of calves. BAdV was the only virus detected in the saliva samples of cows. Another study has already shown that BVDV can be detected in saliva samples, which highlights the importance of the examined sample material [15]. Using the newly established saliva sampling systems, saliva was obtained easily and without stress for the animals. In contrast to sampling with deep nasal swabs, the animals do not have to be restrained. The calves showed especially great interest and chewed on the sampling material independently, so that a large amount of saliva could be collected. Non-invasive saliva collection from the individual cows proved to be more challenging compared to the calves, as they reacted more anxiously to the new sampling system. In the groups, the cows accepted the saliva sampling system well and chewed on the cotton rolls so that enough saliva could be obtained. Another advantage of saliva as a sample material is the easy handling of the sampling system, as it can be used directly by farmers in the future.

BAdV was the most frequently detected virus in deep nasal swabs (35.0%, *n* = 60) and in saliva samples (31.25%, *n* = 48). If only the calves are considered, the detection rate for BAdV is even higher at 58.06% (*n* = 31) in deep nasal swabs and 63.64% (*n* = 22) in the saliva samples. The sequencing results identified BAdV-3 in the positive samples. BAdV-3 is an important respiratory pathogen in cattle and is frequently involved in BRD [23]. Infections with adenoviruses are associated with respiratory [24] and gastrointestinal [25] symptoms, although asymptomatic courses have been described. Calves experimentally infected with BAdV-3 showed pyrexia, hyperpnea, dyspnea, anorexia and, in one animal, mild diarrhea [24].

BoGHV6 was detected in one pooled sample of deep nasal swabs from five cows (20.0%, *n* = 5). Although BoGHV6 is endemic in cattle in Europe with a high prevalence of 32% [26], its clinical relevance is discussed controversially. In a single case, it has been associated with interstitial pneumonia [27], but its role in the development of diseases remains uncertain. Currently, BoGHV6 is assumed to be a part of the virome rather than a pathogen in cattle [28].

BRSV and BPIV3, both important pathogens within BRD, were detected in only a few samples, as shown in Table 6 for deep nasal swabs and in Table 7 for saliva samples. This is most likely due to the high vaccination rates of the involved farms, as vaccination is the best preventive measure to control these two viruses [29].

IAV was not detected in any of the samples tested, whereas IDV was detected in the deep nasal swab of one cow (4.17%, *n* = 24). As the first influenza virus in cattle, IDV was discovered in 2011 and has since been described worldwide [8]. Metagenomic studies showed an association between the detection of IDV and the development of BRD [23].

In the pooled samples from the calf groups, BoCV was detected in 66.67% (*n* = 6) of the deep nasal swabs and in 50.0% (*n* = 4) of the saliva samples. These results are consistent with other studies showing that the virus is commonly present in calves. A recent study across 125 dairy farms in Europe found BoCV in 80% of herds, with 24% of neonatal calves and 23% of weaned calves testing positive within the herds [30]. BoCV causes respiratory and enteric infections in cattle and is associated with different clinical syndromes, like BRD, calf diarrhea and winter dysentery with hemorrhagic diarrhea in adults [31].

Both BRAV and BRBV were frequently detected in calf samples, which is very interesting as there is very little data available to date. BRAV was detected in pooled samples of deep nasal swabs (33.33%, *n* = 6) and saliva (50.0%, *n* = 4) from two groups of calves each. BRBV was also detected in the samples from individual calves at 32.0% (*n* = 25) in deep nasal swabs and at 11.11% (*n* = 18) in saliva. In both matrices (deep nasal swab *n* = 6, saliva *n* = 4) of the sampled calf groups, BRBV was detected in 50.0% of the samples. BRBV infection was shown to be highly prevalent in acute BRD samples with a detection rate of 40.8% (*n* = 49) in deep nasal swabs from cattle with respiratory signs in the USA [32]. The increased use of next-generation sequencing in recent years has contributed to the assumption that bovine rhinitis viruses play a clinical role in BRD [23,33], as BRBV infection has already been demonstrated in the upper respiratory tract [32]. Future research is needed on bovine rhinitis viruses, particularly their etiological involvement and pathogenesis in relation to BRD.

Although bacterial pathogens are recognized as contributing factors to BRD, the current study focused on the detection of respiratory viruses. Bacterial evaluation was not conducted. This limitation should be considered.

Due to the relatively small number of samples, it is difficult to make general statements regarding the agreement between the two matrices, deep nasal swabs and saliva. Overall, it was only possible to collect both sample matrices from 38 individual animals and 9 groups. As shown in Figure 4, there are only a few positive virus detections in both matrices, which limits the evaluation. However, especially in the groups, there is a percentage agreement for positive virus detections in both matrices of 22.22% for BRAV and BRBV and of 33.33% for BAdV and BoCV (*n* = 9). These results suggest that saliva may be a suitable sample material, especially as a diagnostic tool for sampling larger groups. Further studies with larger numbers of animals and matching samples are needed.

The most common discrepancy between the two matrices was that virus was detected in the deep nasal swab but not in the saliva sample. Hence, detection of viral BRD pathogens in deep nasal swabs is slightly more sensitive than in saliva. BPIV3 in individuals and BRSV in groups could, in part, only be detected in saliva, reflecting the complexity of diagnosis. These results highlight the diagnostic challenges of BRD, as samples are usually taken from one location. Since viruses replicate at different sites in the respiratory tract, taking a single sample may limit the accurate diagnosis of the involved pathogens.

## 5. Conclusions

Saliva is a promising sample material for the detection of respiratory pathogens in cattle. Important viruses such as BAdV, BRSV, BPIV3, BoCV, BRAV and BRBV, which are part of the BRD complex, have been successfully detected in saliva samples. Simple and stressless sampling with the novel saliva sampling system promotes the protection and welfare of animals in the cattle industry.

## Figures and Tables

**Table 1:** Overview of viruses involved in BRD, classified according to current ICTV taxonomy. For each virus, the family, genus, species, virus name and abbreviation (Abbrev.) are listed.

Family	Genus	Species	Virus Name	Abbrev.
*Pneumoviridae*	*Orthopneumovirus*	*Orthopneumovirus bovis*	Bovine Respiratory Syncytial Virus	BRSV
*Paramyxoviridae*	*Respirovirus*	*Respirovirus bovis*	Bovine Parainfluenza Virus 3	BPIV3
*Orthoherpesviridae*	*Varicellovirus*	*Varicellovirus bovinealpha1*	Bovine Aalphaherpesvirus 1	BoAHV1
*Coronaviridae*	*Betacoronavirus*	*Betacoronavirus gravedinis*	Bovine Coronavirus	BoCV
*Adenoviridae*	*Barthadenovirus*	*Barthadenovirus bosquartum*	Bovine Adenovirus 4, 5 and 8	BAdV-4/5/8
		*Barthadenovirus bosseptimum*	Bovine Adenovirus 7	BAdV-7
		*Barthadenovirus bossextum*	Bovine Adenovirus 6	BAdV-6
	*Mastadenovirus*	*Mastadenovirus bosprimum*	Bovine Adenovirus 1	BAdV-1
		*Mastadenovirus bostertium*	Bovine Adenovirus 3	BAdV-3
		*Mastadenovirus bosdecimum*	Bovine Adenovirus 10	BAdV-10
*Orthomyxoviridae*	*Deltainfluenzavirus*	*Deltainfluenzavirus influenzae*	Influenza D Virus	IDV
*Orthomyxoviridae*	*Alphainfluenzavirus*	*Alphainfluenzavirus influenzae*	Influenza A Virus	IAV
*Picornaviridae*	*Aphthovirus*	*Aphthovirus bogeli*	Bovine Rhinitis A Virus 1	BRAV-1
			Bovine Rhinitis A Virus 2	BRAV-2
		*Aphthovirus reedi*	Bovine Rhinitis B Virus 1, 2, 3, 4 and 5	BRBV-1/2/3/4/5

**Table 2:** Summary of planned sample collection for each of the five farms.

Group of Animals	Sample Material	
	Deep Nasal Swab	Saliva
5× Individual cows	5× Deep nasal swabs (EYDAM)	5× Saliva sampling system for cows
One group of cows	5× random Deep nasal swabs (EYDAM) =pooled to one sample	2× Saliva sampling system for cows =pooled to one sample
5× Individual calves	5× Deep nasal swabs with UTM™	5× Saliva sampling system for calves
One group of calves	5× random Deep nasal swabs with UTM™ =pooled to one sample	2× Saliva sampling system for calves =pooled to one sample

**Table 3:** Primer sequences used for detection of bovine respiratory viruses in deep nasal swabs and saliva samples. Unless otherwise stated, the PCR and RT-PCR kits are from Thermo Fisher Scientific, Waltham, MA, USA.

Target Virus	Target Gene	PCR Method	PCR Kit	Primer Name	Primer Sequence (5′-3′)	Product Size (bp)	Reference
Adenovirus	DNA polymerase gene	consensus nested PCR	DreamTaq DNA Polymerase	pol Fouter	TNMGNGGNGGNMGNTGYTAYCC	320	[16]
				pol Router	GTDGCRAANSHNCCRTABARNGMRTT		
				pol Finner	GTNTWYGAYATHTGYGGHATGTAYGC		
				pol Rinner	CCANCCBCDRTTRTGNARNGTRA		
Herpesvirus	DNA polymerase gene	consensus nested PCR	DreamTaq DNA Polymerase	KG1	GTCTTGCTCACCAGNTCNACNCCYTT	210	[17]
				DFA	GAYTTYGCNAGYYTNTAYCC		
				ILK	TCCTGGACAAGCAGCARNYSGCNMTNAA		
				TGV	TGTACCTCGGTGTAYGGNTTYACNGGNGT		
				IYG	CACAGAGTCCGTRTCNCCRTACAT		
BRSV	G gene	one-step nested RT-PCR	SuperScript™ III One-Step RT-PCR/Platinum™ Taq High Fidelity DNA-Polymerase and Platinum™ Taq DNA-Polymerase High Fidelity	B5A	CCACCCTAGCAATGATAACCTTGAC	371	[18]
				B6A	AAGAGAGGATGCYTTGCTGTGG		
				B7A	CATCAATCCAAAGCACCACACTGTC		
				B8	GCTAGTTCTGTGGTGGATTGTTGTC		
BPIV3	M gene	one-step RT-PCR	SuperScript™ III One-Step RT-PCR/Platinum™ Taq High Fidelity DNA-Polymerase	M1	AGTGATCTAGATGATGATCCA	328	[19]
				M2	GTTATTGATCCAATTGCTGT		
IAV	M gene	realtime RT-PCR	RevertAid H Minus Reverse Transcriptase and QuantiTect^®^ SYBR^®^ Green PCR Kit (QIAGEN, Hilden, Germany)	Uni 12	AGCAAAAGCAGG	149	[20,21]
				M1F	GATGTYTTTGCAGGRAAGAAC		
				M2R	AABCGTCTACGCTGCAGTCC		
IDV	PB1 gene	one-step RT-PCR	SuperScript™ III One-Step RT-PCR/Platinum™ Taq High Fidelity DNA-Polymerase	F	TGGATGGAGAGTGCTGCTTC	110	[8]
				R	GCCAATGCTTCCTCCCTGTA		
BoCV	N gene	one-step RT-PCR	SuperScript™ III One-Step RT-PCR/Platinum™ Taq High Fidelity DNA-Polymerase	F	TTGGATCAAGATTAGAGTTGGC	237	[22]
				R	CCTTGTCCATTCTTCTGACC		
BRAV	3D polymerase gene	one-step nested RT-PCR	SuperScript™ III One-Step RT-PCR/Platinum™ Taq High Fidelity DNA-Polymerase and Platinum™ Taq DNA-Polymerase High Fidelity	F	GCCGTGTTCTCGGAYGAG	232	this study
				R1	TARGCAACCATMACATARTCRTC		
				R2	TTRAARTCAAAATCRTGGTCYGA		
BRBV	polyprotein gene	one-step RT-PCR	SuperScript™ III One-Step RT-PCR/Platinum™ Taq High Fidelity DNA-Polymerase	F	TACTYGGWCAYACYATAACACC	294	this study
				R	ACCTGTATGAYGGWATCTCRAAACT		

F: forward primer; R: reverse primer.

**Table 4:** Designed primer sets for BRAV and BRBV.

**Target** **Virus**	**Target Gene**	**Primer** **Name**	**Primer Sequence (5′-3′)**	**Position in** **Nucleotide**	**Reference** **Sequence (GenBank Acc.-No.)**
BRAV	3D polymerase gene	F	GCCGTGTTCTCGGAYGAG	6615-6632	NC_038303.1
		R 1	TARGCAACCATMACATARTCRTC	6825-6847	
		R 2	TTRAARTCAAAATCRTGGTCYGA	6870-6892	
BRBV	polyprotein gene	F	TACTYGGWCAYACYATAACACC	7185-7206	NC_010354.1
		R	ACCTGTATGAYGGWATCTCRAAACT	7454-7478	

F: forward primer; R: reverse primer.

**Table 5:** Summary of actual sample collection, which deviates partly from the planned number.

	Cows				Calves			
	Individuals		Groups		Individuals		Groups	
	Swab ^1^	Saliva ^2^	Swab ^1^	Saliva ^2^	Swab ^3^	Saliva ^4^	Swab ^3^	Saliva ^4^
Farm 1	5	2	5 (1)	2 (1)	5	1	5 (1)	2 (1)
Farm 2	4	5	5 (1)	2 (1)	5	3	5 (1)	0
Farm 3	5	5	5 (1)	1 (1)	5	4	5 (1)	0
Farm 4	5	4	5 (1)	2 (1)	5	5	5 (1)	2 (1)
Farm 5	5	5	5 (1)	2 (1)	5	5	6 (2)	2 (2)
Total	24	21	25 (5)	5 (5)	25	18	26 (6)	6 (4)

^1^ deep nasal swab; ^2^ cow saliva sampling system; ^3^ deep nasal swab; ^4^ calf saliva sampling system; (x) number of pools.

**Table 6:** Virus results in deep nasal swabs.

Group of Animals	BAdV	BoHV	BRSV	BPIV3	IAV	IDV	BoCV	BRAV	BRBV
Individual Cows (*n* = 24)	8.33% [2/24]	0.0% [0/24]	0.0% [0/24]	0.0% [0/24]	0.0% [0/24]	4.17% [1/24]	0.0% [0/24]	0.0% [0/24]	0.0% [0/24]
Cow Groups (*n* = 5)	20.0% [1/5]	20.0% [1/5]	0.0% [0/5]	0.0% [0/5]	0.0% [0/5]	0.0% [0/5]	0.0% [0/5]	0.0% [0/5]	0.0% [0/5]
Individual Calves (*n* = 25)	52.0% [13/25]	0.0% [0/25]	4.0% [1/25]	8.0% [2/25]	0.0% [0/25]	0.0% [0/25]	0.0% [0/25]	0.0% [0/25]	32.0% [8/25]
Calf Groups (*n* = 6)	83.33% [5/6]	0.0% [0/6]	0.0% [0/6]	0.0% [0/6]	0.0% [0/6]	0.0% [0/6]	66.67% [4/6]	33.33% [2/6]	50.0% [3/6]
Total (*n* = 60)	35.0% [21/60]	1.67% [1/60]	1.67% [1/60]	3.33% [2/60]	0.0% [0/60]	1.67% [1/60]	6.67% [4/60]	3.33% [2/60]	18.33% [11/60]

**Table 7:** Virus results in saliva samples.

**Group of Animals**	**BAdV**	**BoHV**	**BRSV**	**BPIV3**	**IAV**	**IDV**	**BoCV**	**BRAV**	**BRBV**
Individual Cows (*n* = 21)	4.76% [1/21]	0.0% [0/21]	0.0% [0/21]	0.0% [0/21]	0.0% [0/21]	0.0% [0/21]	0.0% [0/21]	0.0% [0/21]	0.0% [0/21]
Cow Groups (*n* = 5)	0.0% [0/5]	0.0% [0/5]	0.0% [0/5]	0.0% [0/5]	0.0% [0/5]	0.0% [0/5]	0.0% [0/5]	0.0% [0/5]	0.0% [0/5]
Individual Calves (*n* = 18)	61.11% [11/18]	0.0% [0/18]	0.0% [0/18]	5.56% [1/18]	0.0% [0/18]	0.0% [0/18]	0.0% [0/18]	0.0% [0/18]	11.11% [2/18]
Calf Groups (*n* = 4)	75.0% [3/4]	0.0% [0/4]	25.0% [1/4]	0.0% [0/4]	0.0% [0/4]	0.0% [0/4]	50.0% [2/4]	50.0% [2/4]	25.0% [1/4]
Total (*n* = 48)	31.25% [15/48]	0.0% [0/48]	2.08% [1/48]	2.08% [1/48]	0.0% [0/48]	0.0% [0/48]	4.17% [2/48]	4.17% [2/48]	8.33% [4/48]

**Table 8:** Comparison of deep nasal swabs and saliva samples of the 38 individual animals and the 9 groups, of which both sample matrices were available. The individual results for each virus were checked for agreement and grouped in terms of positive result in deep nasal swab and saliva sample (+/+), negative result in deep nasal swab and saliva sample (−/−), positive result in deep nasal swab and negative result in saliva sample (+/−) and negative result in deep nasal swab and positive result in saliva sample (−/+). The sum of the matching results (+/+ and −/−) is also shown. The results are presented as percentages.

Virus	Individual Animals (*n* = 38)					Groups (*n* = 9)				
	+/+	−/−	Sum	+/−	−/+	+/+	−/−	Sum	+/−	−/+
BAdV	26.32%	60.53%	86.86%	7.89%	5.26%	33.33%	55.56%	88.89%	11.11%	0.0%
BoHV	0.0%	100.0%	100.0%	0.0%	0.0%	0.0%	88.89%	88.89%	11.11%	0.0%
BRSV	0.0%	97.37%	97.37%	2.63%	0.0%	0.0%	88.89%	88.89%	0.0%	11.11%
BPIV3	0.0%	92.11%	92.11%	5.26%	2.63%	0.0%	100.0%	100.0%	0.0%	0.0%
IAV	0.0%	100.0%	100.0%	0.0%	0.0%	0.0%	100.0%	100.0%	0.0%	0.0%
IDV	0.0%	97.37%	97.37%	2.63%	0.0%	0.0%	100.0%	100.0%	0.0%	0.0%
BoCV	0.0%	100.0%	100.0%	0.0%	0.0%	33.33%	55.56%	88.89%	11.11%	0.0%
BRAV	0.0%	100.0%	100.0%	0.0%	0.0%	22.22%	77.78%	100.0%	0.0%	0.0%
BRBV	5.26%	78.95%	85.21%	15.79%	0.0%	11.11%	77.78%	88.89%	11.11%	0.0%

## Data Availability

Data is contained within the article.

## Abbreviations

The following abbreviations are used in this manuscript:

BRD	Bovine Respiratory Disease
PCR	Polymerase Chain Reaction
Abbrev.	Abbreviation
RT-PCR	Reverse Transcription Polymerase Chain Reaction
BRSV	Bovine Respiratory Syncytial Virus
BPIV3	Bovine Parainfluenzavirus Type 3
BoAHV1	Bovine Alphaherpesvirus 1
BoHV	Bovine Herpesvirus
BoCV	Bovine Coronavirus
BAdV	Bovine Adenovirus
BAdV-4	Bovine Adenovirus 4
BAdV-5	Bovine Adenovirus 5
BAdV-8	Bovine Adenovirus 8
BAdV-7	Bovine Adenovirus 7
BAdV-6	Bovine Adenovirus 6
BAdV-1	Bovine Adenovirus 1
BAdV-3	Bovine Adenovirus 3
BAdV-10	Bovine Adenovirus 10
IDV	Influenza D Virus
IAV	Influenza A Virus
BRAV	Bovine Rhinitis A Virus
BRAV-1	Bovine Rhinitis A Virus 1
BRAV-2	Bovine Rhinitis A Virus 2
BRBV	Bovine Rhinitis B Virus
BRBV-1	Bovine Rhinitis B Virus 1
BRBV-2	Bovine Rhinitis B Virus 2
BRBV-3	Bovine Rhinitis B Virus 3
BRBV-4	Bovine Rhinitis B Virus 4
BRBV-5	Bovine Rhinitis B Virus 5
BVDV	Bovine Viral Diarrhea Virus
BoGHV6	Bovine Gammaherpesvirus 6
ddH_2_O	Double-Distilled H_2_O
